# NSG-Mice Reveal the Importance of a Functional Innate and Adaptive Immune Response to Overcome RVFV Infection

**DOI:** 10.3390/v14020350

**Published:** 2022-02-08

**Authors:** Lukas Mathias Michaely, Melanie Rissmann, Markus Keller, Rebecca König, Felicitas von Arnim, Martin Eiden, Karl Rohn, Wolfgang Baumgärtner, Martin Groschup, Reiner Ulrich

**Affiliations:** 1 Department of Pathology, University of Veterinary Medicine Hannover, Foundation, Bünteweg 17, 30559 Hannover, Germany; lukas.mathias.michaely@tiho-hannover.de; 2 Center for Systems Neuroscience Hannover, University of Veterinary Medicine Hannover, Foundation, Bünteweg 2, 30559 Hannover, Germany; 3 Institute of Novel and Emerging Infectious Diseases, Friedrich-Loeffler-Institut, Südufer 10, 17493 Greifswald-Insel Riems, Germany; m.rissmann@erasmusmc.nl (M.R.); markus.keller@fli.de (M.K.); rebecca.j.koenig@gmail.com (R.K.); felicitas.von_Arnim@fli.de (F.v.A.); martin.eiden@fli.de (M.E.); martin.groschup@fli.de (M.G.); 4 Institute for Biometry, Epidemiology and Information Processing, University of Veterinary Medicine Hannover, Foundation, Bünteweg 2, 30559 Hannover, Germany; karl.rohn@tiho-hannover.de; 5 Institute of Veterinary-Pathology, Faculty of Veterinary Medicine, Leipzig University, 04103 Leipzig, Germany; reiner.ulrich@vetmed.uni-leipzig.de; 6 Department of Experimental Animal Facilities and Biorisk Management, Friedrich-Loeffler-Institut, Südufer 10, 17493 Greifswald-Insel Riems, Germany

**Keywords:** Rift Valley fever, immunodeficiency, innate immunity, adaptive immunity, virus persistence

## Abstract

Rift Valley fever (RVF) is a zoonotic disease caused by RVF Phlebovirus (RVFV). The RVFV MP-12 vaccine strain is known to exhibit residual virulence in the case of a deficient interferon type 1 response. The hypothesis of this study is that virus replication and severity of lesions induced by the MP-12 strain in immunocompromised mice depend on the specific function of the disturbed pathway. Therefore, 10 strains of mice with deficient innate immunity (B6-IFNAR^tmAgt^, C.129S7(B6)-Ifng^tm1Ts^/J, B6-TLR3^tm1Flv^, B6-TLR7^tm1Aki^, NOD/ShiLtJ), helper T-cell- (CD4^tm1Mak^), cytotoxic T-cell- (CD8^atm1Mak^), B-cell- (Igh-J^tm1Dhu^N?+N2), combined T- and B-cell- (NU/J) and combined T-, B-, natural killer (NK) cell- and macrophage-mediated immunity (NOD.Cg-Prkdc^scid^Il2rg^tm1WjI^/SzJ (NSG) mice) were subcutaneously infected with RVFV MP-12. B6-IFNAR^tmAgt^ mice were the only strain to develop fatal disease due to RVFV-induced severe hepatocellular necrosis and apoptosis. Notably, no clinical disease and only mild multifocal hepatocellular necrosis and apoptosis were observed in NSG mice, while immunohistochemistry detected the RVFV antigen in the liver and the brain. No or low virus expression and no lesions were observed in the other mouse strains. Conclusively, the interferon type 1 response is essential for early control of RVFV replication and disease, whereas functional NK cells, macrophages and lymphocytes are essential for virus clearance.

## 1. Introduction

Rift Valley fever (RVF) is a zoonotic disease caused by Rift Valley fever virus (RVFV) that is endemic in many regions of Africa and Arabia [1,2]. It poses a threat to many host species including cattle, goats, sheep and humans [2,3,4].

RVFV infection causes massive abortion storms and high mortality rates in neonatal ruminants, which are symptomatic of RVF and result in devastating economic losses of local ruminants [5,6]. Furthermore, the disease affects humans, especially those in close contact with animals [7,8]. While most human cases present as subclinical flu-like symptoms, severe or even fatal complications may occur, and the estimated case-fatality rate varies between 0.5 and 2% [9]. These complications include severe hemorrhagic fever, jaundice or a late-onset retinitis and encephalitis [3,4,9]. In addition to impactful outbreaks in Africa, opportunities of RVF introduction into Europe and North America include accidental emergence via animal trading, the spread of mosquito vectors during the course of global warming or bioterrorist attack scenarios [2,4,10]. Because of these threats, RVFV has been named an “agent of concern” by the United States Department of Agriculture (USDA) and the Center for Disease Control and Prevention (CDC) [4]. It is under surveillance by the International Organization for Animal Health (OIE) as well as being a subject of ongoing scientific interest [11,12,13].

As with many viral diseases, the best countermeasure against RVF is an efficient vaccination of susceptible hosts [14,15]. However, no human or veterinary vaccines have been fully licensed in Europe and the United States so far, and there is only limited and often unregulated use of live vaccines, including the RVFV strains MP-12, Clone13 or Smithburn in endemic areas [14,15]. Thus, promising or already field-tested vaccines represent an ongoing target of scientific investigations [14,15]. The development, improvement and investigation of vaccines rely on the understanding of RVFV pathogenesis and immunopathology [15,16,17]. Both are systemic processes and require detailed studies in animal models [18,19]. Besides the traditional hosts such as ruminants, studies in rodents, including susceptible mouse strains, and exotic animals such as non-human primates or toads have been performed [18,19,20,21,22].

In susceptible mice, RVF exhibits a biphasic disease course; after an initial phase characterized by a strong viremia and severe hepatitis, late-onset encephalitis may occur [20,21]. After infection of the host, RVFV is able to replicate within dendritic cells and macrophages [23]. Following replication within macrophages and dendritic cells, RVFV exhibits viremic spread and a strong tropism toward Kupffer cells and hepatocytes, in which it causes multifocal apoptosis and necrosis [17,21,24]. After this acute course of the disease, viral spread into the central nervous system (CNS), most likely along the olfactory epithelium and ascending nerves, can occur as a secondary complication in mice [20,21,25]. The main protection against this course of RVFV infection is provided by the innate immune response [16,26,27,28].

RVFV triggers the innate immune response by activation of different pattern recognition receptors [23]. While the exact receptors are unknown, Toll-like-receptors (TLR) 3 and 7 are expressed within macrophages and dendritic cells, respectively, and detect viral RNA [29]. They activate the TRIF/TRAM (TLR 3)- or MyD88 (TLR 7)-dependent pathways and thereby induce the type I interferon (IFN) response [29]. Thus, they play a key role as pattern recognition receptors in many viral infections, e.g., the RVFV-related Punta Toro virus for which was shown that TLR 3 could even induce detrimental effects due to overstimulation [30,31,32]. Furthermore, it has been shown that genetic polymorphisms of TLR 7 correlate with RVF severity in humans; thus, it can be assumed that TLR 3 and 7 are relevant elements of RVFV recognition [33,34]. The interferon response is counteracted by RVFV via its major pathogenicity factors, the non-structural proteins s (NSs) and m (NSm) [24,35,36,37,38,39,40,41,42]. If successfully mobilized, the IFN type I system, consisting of IFN α and IFN β that bind to the IFN-α/β receptor (IFNAR), induces a wider immune reaction by recruiting effector cells such as lymphocytes to virus-infected cells, which also results in further apoptosis of infected hepatocytes [26,27]. The effector cells lead to an adaptive immune response, with T-lymphocytes adding IFN γ to the interleukin environment and thereby recruiting more macrophages [43]. Moreover, CD4^+^ T lymphocytes and antigen-presenting cells, including dendritic cells and macrophages, prime B-lymphocytes lead to the production of immunoglobulins (Ig) and, in the case of survival, the development of memory cells [44]. This development of antibodies and the immunological memory against RVFV leads to successful control and elimination of the virus and a long-term immune competence to re-infection [45]. Besides these mechanisms, CD8^+^ T-lymphocytes add to the cellular immunity by eliminating infected cells [46].

While some of the aforementioned aspects, e.g., the role of dendritic cells and macrophages as the first location of viral replication, are well-known, other aspects, e.g., the contribution of the different T-cell subtypes within the adaptive immune response, require further investigations; current research efforts focus on the detailed understanding of RVFV pathogenesis and immunogenesis [13]. The hypothesis of this study is that virus replication and severity of lesions induced by the MP-12 strain in immunocompromised mice depend on the specific function of the disturbed pathway. Therefore, the aim is to investigate the clinical disease, viral spread and lesion profile in mouse strains deficient in IFN type 1 signaling (B6-IFNAR^tmAgt^), IFN type 2 signaling (C.129S7(B6)-Ifng^tm1Ts^/J) Toll-like receptor 3 and 7 mediated innate immunity (B6-TLR3^tm1Flv^, B6-TLR7^tm1Aki^), defective natural killer (NK) cells and macrophages (NOD/ShiLtJ), helper T lymphocytes (CD4^tm1Mak^), cytotoxic T lymphocytes (CD8a^tm1Mak^), B lymphocytes (Igh-J^tm1Dhu^N?+N2), T- and B lymphocytes (*Foxn1^nu-/-^*) and combined NK-cells, macrophages and T and B lymphocytes (NOD.Cg-Prkdc^scid^Il2rg^tm1WjI^/SzJ). These differently impaired mice were chosen to evaluate single aspects of the immune response during RVFV infection.

## 2. Materials and Methods

### 2.1. Virus

RVFV strain MP-12 propagation follows previously published protocols [47]. Briefly, it was propagated on Vero-76 cells in Dulbecco’s Modified Eagle’s Medium (DMEM, #DMEM-HXA, Capricorn Scientific GmbH, Ebsdorfergrund, Germany)/2% fetal bovine serum (FBS; #FBS-HI-12A FBS, Capricorn Scientific GmbH, Ebsdorfergrund, Germany) under environmental conditions of 37 °C and a CO_2_ content of 5%. Supernatant of infected cells was harvested after three days, with cells showing a cytopathic effect in 80% of cells, and titers were evaluated in a TCID_50_ assay and calculated according to Spearman and Kaerber [48,49].

### 2.2. Mice

All mice (Table 1) were female and five to seven weeks of age. Homozygous B6-IFNAR^tmAgt^, C.129S7(B6)-lfng^tm1/s^/J, B6-TLR3^tm1Flv^, B6-TLR7^tm1Aki^, B6-CD4^tm1Mak^, B6-CD8a^tm1Mak^, C57Bl/6 and BALB/c were bred and provided by the mouse stock of the FLI, Riems, Germany. *Foxn1^nu-/-^* (NUDE) mice as well as heterozygous *Foxn1^nu+/-^* control animals from the same stock were obtained from Jackson Laboratory (Sulzfeld, Germany), homozygous Igh-J^tm1Dhu^N?+N2 mice were obtained from Taconic Biosciences GmbH (Leverkusen, Germany), and homozygous NOD.Cg-Prkdc^scid^Il2rg^tm1WjI^/SzJ (NSG) as well as homozygous NOD/ShiLtJ (NOD) from Charles River Laboratories, Research Models and Services GmbH (Sulzfeld, Germany), respectively.

### 2.3. Infection and Study Design

All animal experiments were conducted in accordance with German animal welfare laws and authorized by the responsible authority (Landesamt für Landwirtschaft, Lebensmittelsicherheit und Fischerei Mecklenburg-Vorpommern, permission LALLF 7221.3-1-038/17).

Group size was determined using a Cox’s proportional hazards model superiority by a margin analysis employing literature-based a priori estimates of the median survival of the different strains [61,62]. Infection groups of six, nine or twelve specific pathogen-free mice as well as placebo groups of six mice of the same strain were randomly divided into three mice per cage (two to four cages per infection group, Table 1). They were kept in ventilated isocages (Tecniplast S.p.A., Buguggiate, Italy) and provided food (Ssniff Spezialdiäten GmbH, Soest, Germany) and water ad libitum. High caloric food from the same supplier was given to the NUDE mice, as they require increased energy intake due to their lack of fur.

After 14 days of acclimatization, the mice were infected subcutaneously in the neck with RVFV MP-12 (TCID_50_: 1.43 ×10^3^, 100 µL DMEM), while placebo groups were mock infected following the same inoculation route with the same amount of virus-free DMEM. All mice were observed for 14 days and daily body weight and clinical signs (Table 2) were noted.

If the animals developed severe clinical signs, defined as a score of 3 in one category or a score of 2 in all categories, or after reaching 14 days post infection (dpi), they were euthanized by isoflurane anesthesia and subsequent cardiac puncture and blood collection. Necropsy was performed and organ samples were collected, including the brain, spleen, thymus (if applicable), liver, heart, kidney and lungs.

### 2.4. RNA Isolation and Reverse Transcription Quantitative Polymerase Chain Reaction

Mouse tissue samples of 3–78 µg (mean 30 µg) liver, spleen and brain were lysed in cell culture medium using the QIAGEN TissueLyser II^®^ (QIAGEN GmbH, Hilden, Germany) and after centrifugation, RNA was isolated using the NucleoMag^®^ VET Kit (Machery & Nagel GmbH & Co. KG, Düren, Germany) and the automated KingFisher™ Flex Purification System (Thermo Scientific, Inc., Waltham, MA, USA). As internal extraction control, an MS2 bacteriophage was added to each sample [63]. The presence of RVFV- and MS2-derived RNA was verified using a primer-probe based quantitative real-time RT-PCR (qRT-PCR), with a detection limit of five copies per reaction [64]. A synthetic RNA calibrator was utilized for quantification [65].

### 2.5. Serum Neutralization Test

Blood samples from mice were centrifuged and serum was tested for its neutralization ability in decreasing dilutions (serum neutralization test, SNT) as described previously [66]. Briefly, 100 TCID_50_ of MP-12 were added to duplicates of serial two-fold diluted (from 1:10 to 1:120) and heat inactivated sera. Following an incubation of 30 min at 37 °C and 5% CO_2_, Vero-76 cells were added to each well. Plates were incubated at 37 °C, 5% CO_2_ for six days. Neutralizing doses of 50%(ND_50_) were expressed as the reciprocal of the serum dilution that still inhibited > 50% of cytopathogenic effect and calculated as described by Spearman and Kaerber [43,44]. The cytopathogenic effect was macroscopically evaluated after fixation with 4% paraformaldehyde and staining with 1% crystal violet.

### 2.6. Histology and Immunohistochemistry

Mouse tissue samples of brain, spleen, liver, heart, kidney and lungs were fixed in 4% neutral buffered paraformaldehyde for 21 days, were cut according to Registry of Industrial Toxicology Animal-data (RITA) trimming guides and were subsequently embedded in paraffin wax [67]. Four µm thick sections were stained with hematoxylin and eosin (HE) stain. In addition, immunohistochemistry (IHC) with a primary antibody targeting RVFV nucleoprotein (Np) was performed on 2 µm sections. Briefly, slides were dewaxed and rehydrated, microwaved in citrate buffer (700 Watt, 20 min, pH: 6.01) and blocked by 1 h incubation with rabbit serum. Thereafter, heat-inactivated serum from a RVFV-infected sheep was applied overnight at 4 °C as a primary antibody, followed by biotinylated rabbit anti-sheep secondary antibodies. ABC Kit Vectastain (PK 6100 Biozol Diagnostica Vertrieb GmbH, Eching, Germany) and AEC Substrat Chromogen Ready to use (K3464 Dako Denmark A/S, Glostrup, Denmark) were applied according to the manufacturer’s instructions, followed by hematoxylin counterstain. RVFV-infected and non-infected cell pellets were used as positive and negative controls, respectively. Details of the staining protocols have been published previously [47,68,69].

Sections were evaluated by qualitative description of HE stained lesions and manual cell count of immunohistochemically labeled cells per 30 high power fields (1 HPF = 0.159 mm^2^; using a Zeiss standard light microscope (Carl Zeiss AG, Oberkochen, Germany) with a Zeiss Kpl-W10x/18 ocular and a Zeiss 40/0.65 objective). Semi-quantification of immunohistochemistry was counted as follows: −: no findings; +: up to five positive cells per HPF; ++: five to 20 positive cells per HPF; +++: over 20 positive cells per HPF/diffuse expression of RVFV Np. Pictures were taken with an Olympus BX51 microscope and a DP72 Camera using the manufacturer‘s operating software cellSens, version 1.18 (Olympus Solutions Inc., Tokyo, Japan).

### 2.7. Statistical Analysis

The assumption of normal distribution of RT-qPCR results was rejected using Shapiro–Wilks’ test and visual assessment of the qq plots of the model residuals. For descriptive statistics, measures of location and statistical dispersion were depicted as median and range with min and max values. The survival rate of all RVFV-infected mouse strains and placebo groups was calculated and depicted using the Kaplan–Meier analysis. SNT data were tested for differences between the infected groups and the placebo controls using a Kruskal–Wallis test for independent samples followed by bilateral multiple pairwise comparisons (Dwass, Steel, Critchlow–Fligner method). Figures and statistical workup of the gathered data were performed using the commercially available software Graphpad Prism (GraphPad Software, Inc., San Diego, CA, USA, version 9.0.0) and SAS 9.4 (SAS Institute, Inc. Cary, NC, USA).

## 3. Results

### 3.1. Survival and Clinical Signs

**Figure 1:**
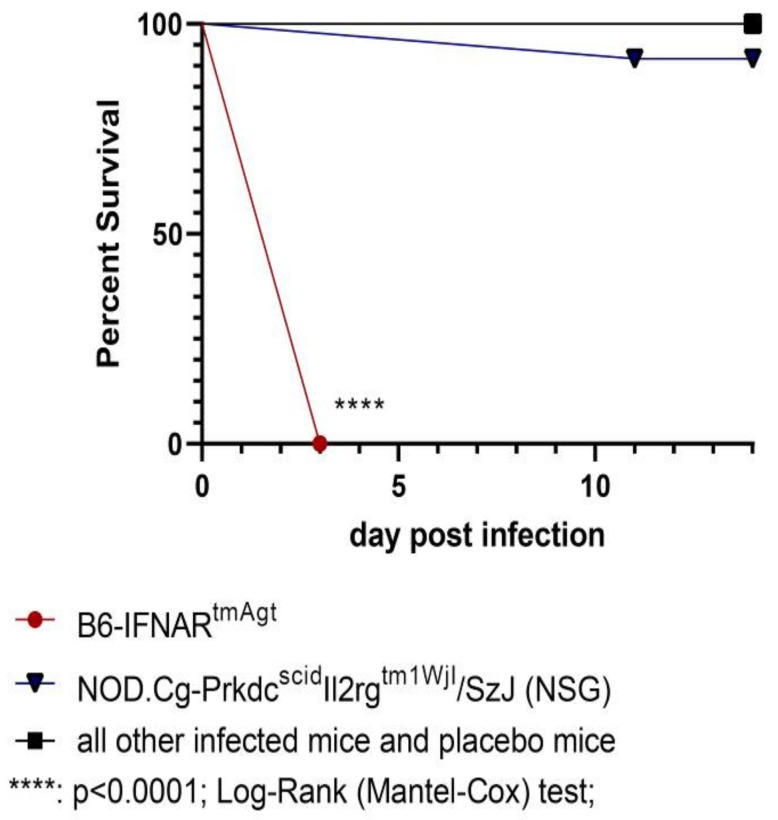
Kaplan–Meier survival curve of all mouse strains infected with RVFV and placebo groups: B6-IFNAR^tmAgt^ mice died or were euthanized three days after subcutaneous RVFV infection. Except for 1/12 (8%) of the NSG mouse, all other mice survived until the end of the study after subcutaneous RVFV infection. The surviving groups are summarized for better readability. RVFV: Rift Valley fever virus.

The RVFV-infected B6-IFNAR^tmAgt^ mice died or had to be euthanized 3 dpi (Figure 1) due to severe clinical signs of disease, including lack of spontaneous or provoked movement, apathy, labored breathing, ruffled fur, hunched posture and final weight loss of over 15% of the original body weight. Furthermore, one RVFV-infected NSG mouse had to be euthanized at 11 dpi due to similar severe clinical signs. All other animals in this group and in the other mouse strains survived until the end of the experiment at 14 dpi and did not show any clinical signs besides mild weight loss (<15%).

### 3.2. Reverse Transcription Quantitative Polymerase Chain Reaction

Detailed RT-qPCR results are presented in Table 3.

**Figure 2:**
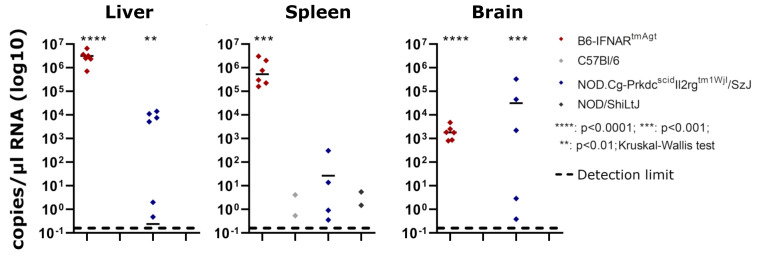
Comparisons of PCR results in B6-IFNAR^tmAgt^ and NOD.Cg-Prkdc^scid^Il2rg^tm1Wjl^/SzJ mice: RT-qPCR results of both mouse strains show significant viral loads in liver, spleen and brain when compared to RVFV-infected wildtype controls (C57Bl/6 or NOD/ShiLtJ mice, respectively).

Briefly, all samples from RVFV-infected B6-IFNAR^tmAgt^ mice (6/6; 100%) that were obtained at 3 dpi yielded high loads of viral RNA with values between 8.03 × 10^2^ to 4.76 × 10^3^ copies/µL RNA (brain), 1.6 × 10^5^ to 3.02 × 10^6^ copies/µL RNA (spleen) and 7.08 × 10^5^ to 6.54 × 10^6^ copies/µL RNA (liver), respectively (Table 3, Figure 2). The group of RVFV-infected NSG mice showed inconsistent results: seven out of twelve (58%) animals exhibited viral RNA in at least one of the three organ samples, and these results ranged from 3.53 × 10^−1^ to 3.29 × 10^5^ copies/µL RNA (Table 3, Figure 2). No viral RNA was found in the remaining animals 5/12 (42%) of this group.

Five of twelve (42%) RVFV-infected B6-TLR7^tm1Aki^ and three of twelve (25%) RVFV-infected C.129S7(B6)-lfng^tm1/s^/J mice, respectively, exhibited small (4.4 × 10^−1^ – 2.54 × 10^1^ copies/µL RNA) amounts of viral RNA in the spleen, and two of twelve (17%) RVFV-infected Igh-J^tm1Dhu^N?+N2 mice exhibited small amounts of viral RNA (<0.1 × 10^0^ copies/µL RNA) in the liver. One of twelve (8%) RVFV-infected B6-CD8a^tm1Mak^ mice, 2/12 (17%) RVFV-infected NOD mice and 3/12 (25%) RVFV-infected BALB/c mice exhibited low viral loads in the spleen (1.47 × 10^0^ to 1 × 10^2^ copies/µL RNA). Furthermore, 3/12 (25%) RVFV-infected C57Bl/6 mice showed viral RNA within the spleen or the brain, respectively (5.41 × 10^−1^ to 2.37 × 10^1^ copies/µL RNA). No viral RNA was found in the other infected mice or the placebo mice.

### 3.3. Serum Neutralization Test

**Figure 3:**
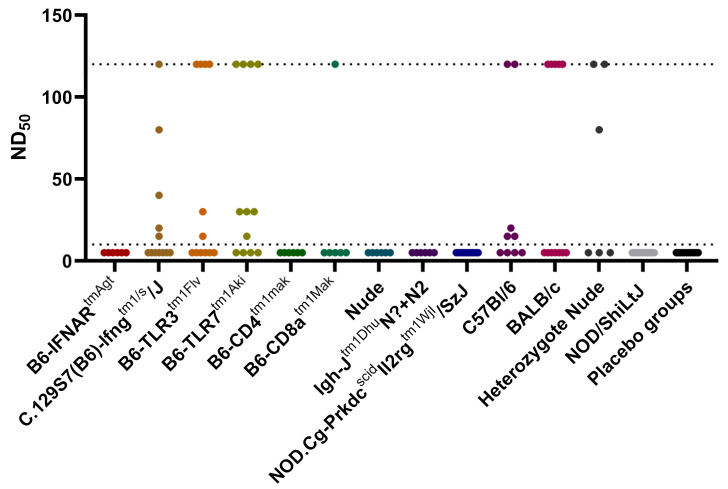
Serum neutralization test results of all mouse strains infected with RVFV and placebo groups: detectable antibody titers in serum samples were measured between 1:10 and 1:120 dilutions. The placebo groups of all mouse strains are summarized for better readability. The detection range is shown as punctuated lines. RVFV: Rift Valley fever virus.

Regarding the SNT results, only a subset of mouse strains survived for 14 days and provided a functional antigen-presenting-cell, T-helper and B-lymphocyte axis (C.129S7(B6)-Ifng^tm1/s^/J, B6-TLR3^tm1Flv^, B6-TLR7^tm1Aki^, B6-CD8a^tm1Mak^, C57Bl/6, BALB/c and heterozygous NUDE). These mouse groups included single or multiple animals showing serum neutralizing activity in dilutions > 1:10 (Figure 3). Statistical analyses revealed a significant difference between the groups (Kruskal–Wallis test *p* < 0.0001). However, the post hoc pairwise comparisons revealed a significant difference only for B6-TLR7^tm1Aki^ versus placebo controls. No seroconversion was observed in the IFNAR^tmAgt^, NOD, B6-CD4^tm1Mak^, Igh-J^tm1Dhu^N?+N2, NUDE or NSG mice.

### 3.4. Histology and Immunohistochemistry

Histology and immunohistochemistry results of B6-IFNAR^tmAgt^ and NSG mice are presented in Table 4.

**Figure 4:**
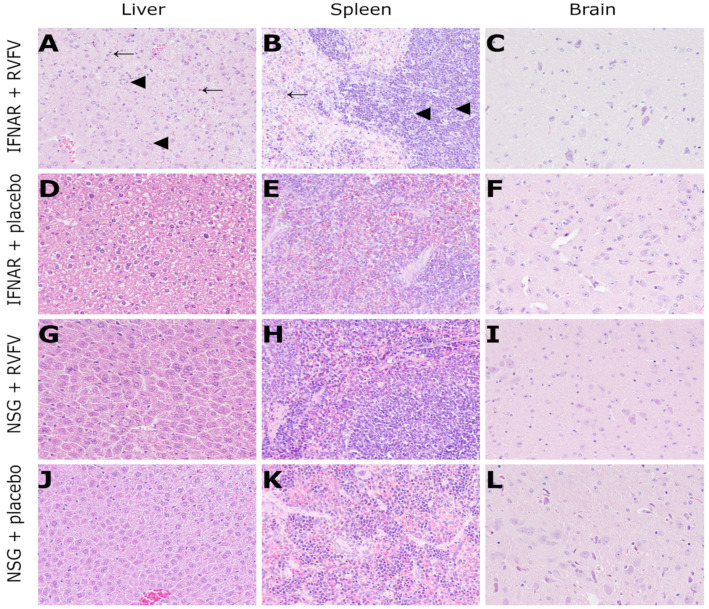
Histology (HE staining, 200x magnification) of RVFV-infected IFNAR mice (3 dpi) and NSG mice (14 dpi): IFNAR mice exhibit severe, multifocal to coalescing hepatocellular necrosis (arrowheads, (**A**)) and apoptosis (arrows, (**A**)) as well as mild lymphocytolysis in the white pulp (arrowheads, (**B**)) and moderate, multifocal necrosis in the red pulp of the spleen (arrow, (**B**)). No lesions were observed in the brain (**C**) and placebo-infected control mice (**D**–**F**). Likewise, no lesions were present in the majority of RVFV-infected (**G**–**I**) or any placebo (**J**–**L**) NSG mice. RVFV: Rift Valley fever virus; dpi: days post infection; IFNAR: B6-IFNAR^tmAgt^ mice. NSG; NOD.Cg-Prkdc^scid^ Il2rg^tm1WjI^/SzJ mice.

RVFV-infected B6-IFNAR^tmAgt^ mice exhibited a severe, diffuse, hepatocellular necrosis characterized by hypereosinophilic cellular debris and nuclear pyknosis as well as karyorhexis. Apoptosis was also present as shown by swollen, eosinophilic hepatocytes (resembling Councilman bodies). Furthermore, multifocal hemorrhages were detected. The same hepatic changes were found to a lesser (mild) degree in two RVFV-infected NSG mice (Figure 4, Table 4). In addition to the liver findings, B6-IFNAR^tmAgt^ mice showed moderate lymphocytolysis in the red pulp of the spleen and mild lymphocytolysis in splenic follicles (Figure 4, Table 4). All other mice, including placebo controls, did not show any lesions. Moreover, the CNS, lung, heart and kidney lacked significant microscopic lesions in all groups.

**Figure 5:**
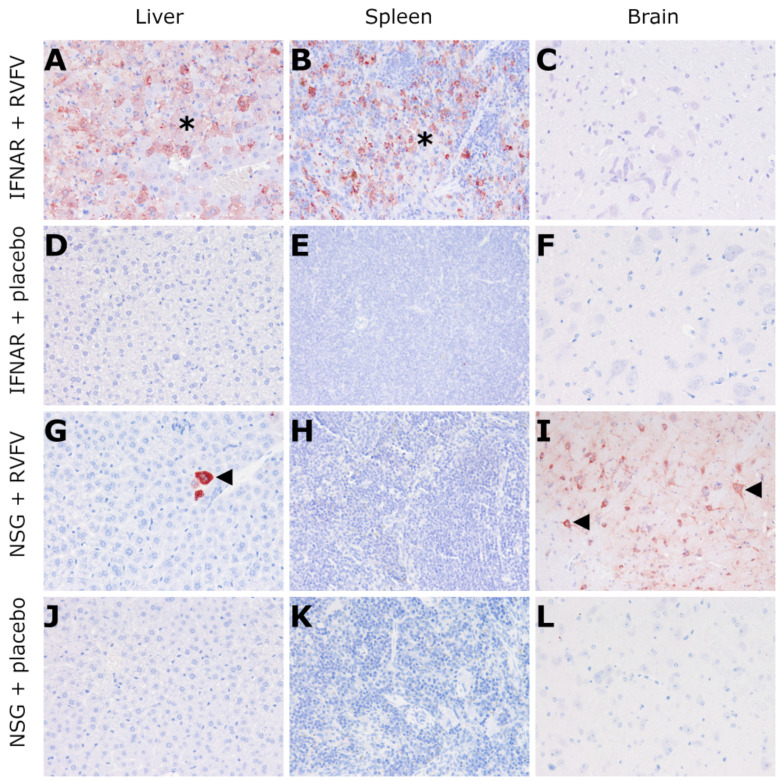
Immunohistochemistry (against RVFV Np, 200x magnification) of RVFV-infected IFNAR mice (3 dpi) and NSG mice (14 dpi): IFNAR mice exhibit multifocal to diffuse expression of RVFV Np in the liver (star (**A**)) and the spleen (star (**B**)) while 3/6 (50%) animals did not show any expression of RVFV Np in the brain (**C**). Control animals are negative for RVFV antigen (**D**–**F**). NSG mice reveal small nests of immunolabeled hepatocytes (arrowhead, (**G**)), no signal in the spleen (**H**) and diffuse expression of RVFV Np in neurons (arrowheads, (**I**)). Control animals are negative (**J**–**L**). RVFV: Rift Valley fever virus; Np: nucleoprotein; dpi: days post infection; IFNAR: B6-IFNAR^tmAgt^ mice; NSG: NOD.Cg-Prkdc^scid^ Il2rg^tm1WjI^/SzJ mice.

**Figure 6:**
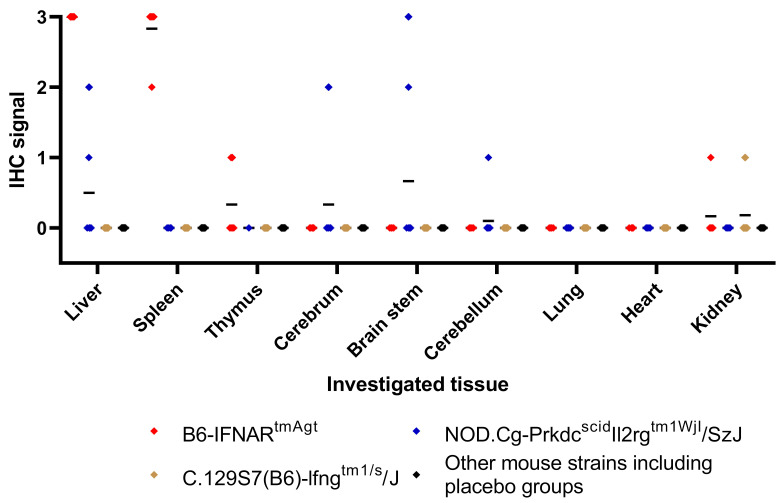
Graphic summary of the semi-quantitative immunohistochemistry evaluation: Each point represents one individual animal and mean values are given (black bars). While B6-IFNAR^tmAgt^ mice exhibit high scores in liver and spleen, NOD.Cg-Prkdc^scid^Il2rg^tm1WjI^/SzJ mice show RVFV Np expression within the CNS in a subset of animals. Furthermore, a single C.129S7(B6)-Ifng^tm1/s^/J mouse exhibited viral antigen expression in the kidney.

Immunohistochemistry revealed RVFV Np in a multifocal to diffuse pattern throughout the liver tissue of RVFV-infected B6-IFNAR^tmAgt^ mice (Figure 5, Table 4). Furthermore, there were abundant amounts of RVFV antigen present within the cytoplasm of sinusoidal macrophages in the spleen, and there were single neurons labeled positive in 3/6 (50%) mice (Figure 5, Table 4). In contrast, RVFV-infected NSG mice exhibited RVFV antigen within the cytoplasm of neurons and small groups of hepatocytes (Figure 5, Table 4). Two and three animals from the RVFV-infected B6-IFNAR^tmAgt^ and NSG mice, respectively, exhibited immunohistochemical labeling of RVFV Np in epithelial cells of kidney tubules and lung bronchioles (Figure 6). However, no histological lesion was associated with these signals. The remaining groups and tissues, including the placebo controls, lacked expression of RVFV Np.

## 4. Discussion

The aim of the present study was to investigate clinical disease, viral spread and lesion profile in mouse strains that are deficient in IFN type 1 signaling (B6-IFNAR^tmAgt^), IFN type 2 signaling (C.129S7(B6)-Ifng^tm1Ts^/J) Toll-like receptor 3 and 7 mediated innate immunity (B6-TLR3^tm1Flv^, B6-TLR7^tm1Aki^), defective natural killer (NK) cells and macrophages (NOD/ShiLtJ), helper T lymphocytes (CD4^tm1Mak^), cytotoxic T lymphocytes (CD8a^tm1Mak^), B lymphocytes (Igh-J^tm1Dhu^N?+N2), T- and B lymphocytes (*Foxn1^nu-/-^*) and combined NK-cells, macrophages and T and B lymphocytes (NOD.Cg-Prkdc^scid^Il2rg^tm1WjI^/SzJ). Surprisingly, no clinical signs, development of virus neutralizing antibodies or associated pathological changes were noticed in NSG mice. Except for B6-IFNAR^tmAgt^ mice, all other investigated knockout mice with impaired innate immunity, such as TLR-deficient B6-TLR7^tm1Aki^ and B6-TLR3^tm1Flv^ mice, or adaptive immunity such as T-cell deficient B6-CD4^tm1Mak^ and B6-CD8a^tm1Mak^ as well as B cell deficient Igh-J^tm1Dhu^N?+N2 mice, were able to control and eliminate RVFV.

In all host species, innate immunity is a key component of early RVFV detection and activation of further effector cells, e.g., macrophages, via release of cellular mediators including IFNs [24,27,34,45,70]. The B6-IFNAR^tmAgt^ mice lack the IFN type I receptor (IFNAR) and showed severe RVF characterized by severe clinical signs, necrotizing hepatitis and virus spread to various organs as described previously [37,50]. However, other knockout mice strains with a deficient innate immunity, including lack of TLR 3, TLR 7 and IFN γ activity, respectively, were characterized by absence of clinical disease, virus antigen expression and lesion development in the investigated organs [52,70,71]. These results show the pathogenicity of the RVFV MP-12 strain in an IFN type 1 deficient immune system [37]. Clinical signs, pathological lesions, immunohistochemistry results and RT-qPCR detection of high amounts of viral RNA in the B6-IFNAR^tmAgt^ mice resemble previously published descriptions of severe RVF in susceptible mice, infected with different RVFV strains including MP-12, and due to their resemblance to human RVF courses, mice have been used as a model species [18,19,20,21,72]. The effect of the other knockouts of the innate immune system, however, appears to be compensated by other mechanisms. IFN γ is the main activator of macrophages, which together with other antigen-presenting cells, initiate the adaptive immune response [73]. However, activation of macrophages can be obtained by other stimuli, e.g., tumor necrosis factor (TNF), which is a substantial component of the interleukin pathway produced by Th17 helper cells [73]. TLR 3 and 7 are pivotal components for RNA detection, and their contributions to the early immune response within viral infection have been shown for multiple viral diseases, e.g., dengue fever [29,74]. TLR activation is another initial step in the immune response triggering cascade, as both receptors recognize double-strained or single-stranded RNA, respectively, and therefore initiate antiviral immune responses [29]. As neither B6-TLR3^tm1Flv^ nor B6-TLR7^tm1Aki^ mice developed clinical RVF, initial recognition of the virus seems to be sufficiently achieved by one of the TLR or another intracellular receptor of viral RNA. Alongside TLR 3 and 7, other receptors of viral RNA, e.g., RIG-I, have been shown to be associated with severe RVF in humans [33]. Therefore, other RNA receptors might represent promising targets of future investigations [13]. RT-qPCR results indicate that mice lacking TLR 7 may require more time for successful virus elimination, as shown by 5/12 mice that exhibited small loads of viral genetic material in the spleen at 14 dpi indicating a less efficient activation of virus clearance mechanisms, e.g., macrophage activation, when lacking TLR 7. This difference in receptor efficacy might be explained by the respective triggered pathways. While TLR 3 triggers an IFN response via a TRIF/TRAM-dependent pathway, TLR 7 achieves this by activating the MyD88/TIRAP pathway [29]. Furthermore, different virus infections highlight the different roles of TLR 3 and 7. Although TLR 3 signaling appears crucial to the immune response against some infections, e.g., herpes simplex virus, the importance of TLR 7 was shown for others, e.g., avian influenza [30,75,76].

In most viral diseases including RVF, the key aspect of the adaptive immunity is the lymphocyte response that includes the production of antibodies and the development of a long-lasting immunity after virus clearance [45]. This protection by RVFV-targeted antibodies has been shown in several studies evaluating RVF vaccination attempts in ruminants [77,78,79]. CD4^+^, CD8^+^, T lymphocyte or B lymphocyte impaired mice were able to control the disease and developed neither clinical disease nor significant viral loads as determined by RT-qPCR and immunohistochemistry. This shows that a full elimination can be achieved by an intact IFN response followed by effector cells, e.g., macrophage activity, the role of which during RVF development was already studied with regard to initial virus spread [80,81]. Furthermore, as the adaptive immune response plays a crucial role in the later course of RVF, a successful elimination of the virus may even be achieved predominantly by the innate immune response as RVFV strain MP-12 is viewed as widely attenuated in IFN competent mice [20,37]. Over and above the deficiency in T- and B-cell-mediated adaptive immunity shared with NUDE mice, NSG mice are also deficient in innate immune pathways; namely, they are deficient in NK cells and their macrophages exhibit functional defects, leading to defective antigen presentation and altered immunoregulation [57,58,59]. They neither develop follicles in spleen and lymph nodes, nor Natural Killer cells, B-cells nor detectable antibody titers in serum samples [54,57]. These mice can be used for cancer or xenograft research studies as they are resistant to lymphoma development and do not show inflammatory responses to xenograft implants [54,57,82]. It seems reasonable, that the combination of defective innate pathways and lacking adaptive immunity in NSG mice as compared to NOD and NUDE mice is responsible for the lack of host control of ongoing virus replication in the NSG mice. Concerning the molecular pathway responsible for reduced or lacking function of NK-cells, the Il2rg^tm1Wjl^ null mutation in NSG mice, which results in the loss of most of the extracellular domain and all of the transmembrane and cytoplasmic domains of the protein, is different from their background strain NOD, which exhibits an Il2^m1^ hypoactive polymorphism [83]. This difference may be the reason for a more profound deficiency in functional NK cells in NSG mice compared to the NOD strain. An increased activation of and a suggested alleviating effect on the early stage of viral infections by NK cells has been shown for various viral infections such as dengue virus, Zika virus, hantavirus or tick-borne encephalitis virus [84]. While their role in systemic RVFV infection remains unclear in detail, a rise of NK cells in liver and lymph nodes of RVFV-infected mice has been observed previously [44]. Therefore, a lack of NK cells could be a contributing factor for the lack of virus clearance in NSG mice and therefore warrants further investigations.

Clinical disease was observed in only one of six NSG mice, although all six animals exhibited viral loads in the RT-qPCR analysis and in four animals, viral loads were detected in the liver, spleen and brain, indicating a viremic phase and infection of the CNS as described during the course of RVF [3,4]. However, RVFV antigen was found in hepatocytes and neurons, while no CNS lesions and only small foci of hepatocellular damage were observed. These results suggest that NSG mice are unable to control viral spread, but at the same time do not regularly succumb to or even develop clinical disease. The IL15-receptor is a heterodimer of the IL-2/IL-15 receptor beta chain (CD122) and the common gamma chain (gamma-C, CD132), the latter being the target of the null mutant in NSG mice [83]. There are no studies on the importance of NK cells in human or ruminant RVF pathogenesis. However, it has been shown that clinically ill Puumala hantavirus-infected humans exhibit a marked increase and activation of NK cells due to an IL15-mediated mechanism [85,86]. This NK overactivation has been suggested to be a factor involved in the pathogenesis of Puumala hanta virus-induced disease, leading to collateral damage on surrounding host cells regardless of their infection status [85,86]. Therefore, it seems reasonable that the lack of NK cells in NSG mice is mediated by a lack of IL2rg-function and may be the reason for both, the lack of MP-12 RVFV clearance as well as an explanation for the lack of lesions and clinical disease in the current study. Further studies are needed to unravel the role of NK-cell mediated mechanisms in the pathogenesis of RVFV-induced hepatitis and encephalitis in other host species such as humans and cattle.

Regarding the applicability of the present results and its conclusions to other host species, two limiting factors must be mentioned. Although mice are an established animal model of RVF and widely used, the present mouse strains are genetically modified and lack certain aspects of their immune responses [18,19,87]. Although the variety of investigated knockout and background strains within the present study allows scientifically sound conclusions with respect to the murine immune system, the importance of different cell types of the immune response, including the NK cell response, may differs in other species, especially humans and ruminants. These aspects still require detailed investigations [13]. Furthermore, the RVFV strain used in the present study is not a wild-type RVFV strain. RVFV MP-12 is attenuated by its heat sensitivity and a combination of several mutations in all three segments of its genome [20,88]. These multiple mutations were derived by repeated mutagenic passages in 5-fluorouracil [89]. However, the main pathogenicity factor, NSs, is only partially attenuated in RVFV MP-12 and retains some of its virulence [90]. Moreover, RVFV MP-12 still has been shown to be lethal to certain, susceptible mouse strains, e.g., IFNAR^-/-^ or STAT1^-/-^ mice [20,37]. Furthermore, its use is allowed under biosafety level (BSL) 2 conditions, and it is even conditionally licensed as a vaccine in the United States [20,37,88]. A non-attenuated wild-type isolate of RVFV may be less likely to show similar results as RVFV MP-12 did in the present study, due to its increased virulence.

During its evaluation as a RVF vaccine, RVFV MP12 was shown to induce seroconversion and protection against re-infection within ruminants and primates [91,92]. The seroconversion was measured in the present study, and although no statistical significance was yielded, a trend toward successful seroconversion in a subset of infected mouse strains was observed. B-cell deficient mice and mice lacking CD4^+^ T-helper cells are naturally unable to produce neutralizing antibodies, and the B6-IFNAR^tmAgt^ succumbed to disease too quickly to develop a detectable adaptive immune response [93]. The remaining mouse strains exhibited inconsistent results ranging from no to high antibody titers with exception of the NOD mice that did not show seroconversion at all, most likely due to their deficiency in antigen presentation and immune regulation [57,58,59].

In summary, the present study shows that a deficiency of IFN type 1 signaling results in fatal RFV disease in mice, whereas a lack of functional NK-cells and combined lack of T- and B-cells as well as defective macrophages is associated with continuous viral replication without development of clinical disease and lesions in the liver and CNS in NSG mice. However, as mice are an established model species but do not represent a natural host to RVF and as an attenuated RVFV strain was used, the results must be interpreted with caution and warrant further investigation regarding their relevance and applicability to humans and ruminants.

## Figures and Tables

**Table 1:** Various mouse strains used for the present investigation and their relevant characteristics.

No.	Designation	Immunological Trait	Number of Infected/Placebo Animals	Origin	References
1	B6-IFNAR^tmAgt^	No IFNAR expression	6/6	FLI	[50]
2	C.129S7(B6)-Ifng^tm1/s^/J	Impaired Interferon γ response and therefore decreased activity of macrophage function	12/6	FLI	[51]
3	B6-TLR3^tm1Flv^	TLR 3 deficiency	12/6	FLI	[52]
4	B6-TLR7^tm1Aki^	TLR 7 deficiency	12/6	FLI	[52]
5	B6-CD4^tm1Mak^	Block of CD4^+^ T-Lymphocyte development and restricted T-helper-cell response	6/6	FLI	[53]
6	B6-CD8a^tm1Mak^	Deficient in functional cytotoxic T-Lymphocytes	6/6	FLI	[54]
7	*Foxn1^nu-/-^* (NUDE)	Lack of thymus and therefore absence of T-Lymphocytes, partial defect in B-cell development	6/6	JAX	[55]
8	Igh-J^tm1Dhu^N?+N2	No B-cell maturation, therefore no IgM or IgG production	6/6	TAC	[56]
9	NOD.Cg-Prkdc^scid^ Il2rg^tm1WjI^/SzJ (NSG)	No lymphocyte maturation (B and T cells), therefore no IgG and extremely low cytotoxic T-cells, deficiency of NK cells, macrophages and dendritic cells, absence of complement C5	12/6	CRL	[54,56,57,58,59]
10	C57Bl/6	Wildtype, background of strain 1, 3-6	9/6	FLI	[20]
11	BALB/c	Wildtype, background of strain 2 and 8	12/6	FLI	[20]
12	*Foxn1^nu+/-^* (NUDE heterozygous)	Heterozygous control for strain 7	6/6	JAX	[55]
13	NOD/ShiLtJ (NOD)	Background of strain 9 (NSG), late-onset spontaneous Autoimmune diabetes mellitus, deficient NK cells, macrophages, dendritic cells and complement component C5	12/6	CRL	[56,58,59,60]

No.: Continuing number for better readability; IFNAR: interferon-α/β receptor; TLR: Toll-like receptor; CD: cluster of differentiation; Ig: immunoglobulin; FLI: Friedrich–Loeffler Institute; NK: natural killer; JAX: Jackson Laboratories; TAC: Taconic biosciences; Wildtype: no genetic alterations; CRL: Charles River Laboratories.

**Table 2:** Clinical score scheme.

Category	Description of Signs	Score
Posture and appearance	Normal posture, smooth fur	0
	Normal posture, ruffled fur	1
	Mildly hunched back, ruffled fur	2
	Severely hunched back, ruffled fur, lack of cleaning	3
Behavior and activity	Curious and alert	0
	Calm, mildly reduced spontaneous movement	1
	Apathy, moderately reduced spontaneous movement, mildly reduced induced movement	2
	Stupor, no spontaneous movement, severely reduced induced movement	3
Body weight	No change >5%	0
	Decrease of 5–15%	1
	Decrease of 15–25%	2
	Decrease of >25%	3

Mice were euthanized after reaching a score of 3 in one category or a score of 2 in all categories.

**Table 3:** RT-qPCR detection of RVFV RNA shown as positive animals/mouse strain and copies/µL RNA in liver, spleen and brain.

Mouse Strain	Liver	Spleen	Brain
B6-IFNAR^tmAgt^	6/6 (7.08 × 10^5^–6.54 × 10^6^)	6/6 (1.6 × 10^5^–3.02 × 10^6^)	6/6 (8.03 × 10^2^–4.76 × 10^3^)
C.129S7(B6)-Ifng^tm1/s^/J	n.d.	3/12 (4.4 × 10^−1^–2.54 × 10^1^)	n.d.
B6-TLR3^tm1Flv^	n.d.	n.d.	n.d.
B6-TLR7^tm1Aki^	n.d.	5/12 (6.28 × 10^−1^–5.94 × 10^0^)	n.d.
B6-CD4^tm1Mak^	n.d.	n.d.	n.d.
B6-CD8a^tm1Mak^	n.d.	1/6 (1 × 10^2^)	n.d.
*Foxn1^nu-/-^* (NUDE)	n.d.	n.d.	n.d.
Igh-J^tm1Dhu^N?+N2	2/6 (4.08 × 10^−3^–8.38 × 10^−2^)	n.d.	n.d.
NOD.Cg-Prkdc^scid^ Il2rg^tm1WjI^/SzJ (NSG)	6/12 (4.74 × 10^−1^–1.41 × 10^4^)	4/12 (3.53 × 10^−1^–3.03 × 10^2^)	6/12 (2.87 × 10^−2^–3.29 × 10^5^)
C57Bl/6	n.d.	2/9 (5.41 × 10^−1^–4.06 × 10^0^)	1/9 (2.37 × 10^1^)
BALB/c	n.d.	3/12 (1.41 × 10^0^–7.05 × 10^0^)	n.d.
*Foxn1^nu+/-^* (heterozygous NUDE)	n.d.	n.d.	n.d.
NOD/ShiLtJ (NOD)	n.d.	2/12 (1.47 × 10^0^–5.43 × 10^0^)	n.d.

Data are presented as follows: number of positive animals/group size (x/x) with range of RVFV RNA copies per µL isolate from the respective organ. IFNAR: interferon-α/β receptor; TLR: Toll-like receptor; CD: cluster of differentiation; n.d.: not detected.

**Table 4:** Summary of histology and immunohistochemistry results of B6-IFNAR^tmAgt^ and NSG mice.

Mouse Strain	Histology	IHC Liver	IHC Spleen	IHC Brain	IHC Heart	IHC Lung	IHC Kidney
B6-IFNAR^tmAgt^	Severe, diffuse, hepatocellular necrosis and apoptosis; Mild to moderate lymphocytolysis in the spleen	+++	+++	+ (3/6) - (3/6)	-	+ (1/6) -(5/6)	+(2/6) -(4/6)
NOD.Cg-Prkdc^scid^ Il2rg^tm1WjI^/SzJ (NSG)	Mild, multifocal, hepatocellular necrosis and apoptosis (2/12); No findings (10/12)	++	-	+++	-	+(2/12) -(10/12)	++(3/12) -(9/12)

RVFV: Rift Valley fever virus. IHC: immunohistochemistry against RVFV Nucleoprotein (Np). -: no findings. +: up to five positive cells per HPF. ++: up to 20 positive cells per HPF. +++: over 20 positive cells per HPF/diffuse expression of RVFV Np. HPF: high power field (0.159 mm^2^). Negative results from other groups are not shown. Animal numbers are given in groups with inconsistent findings.

## Data Availability

The data presented in this study are not publicly available but are available upon reasonable request.
